# Comparison of readmission outcomes and complications between leadless and traditional transvenous pacemakers in older adults: a nationwide readmission analysis of 49852 admission events

**DOI:** 10.1093/europace/euaf268

**Published:** 2025-10-29

**Authors:** Jiaqi He, Keting Liang, Ruijian Huang, Cunhua Su, Jiancheng Zhou, Lingli Wang, Jifang Zhou

**Affiliations:** School of International Business, China Pharmaceutical University, No. 639 Longmian Avenue, Jiangning District, Nanjing, Jiangsu 211198, China; School of International Business, China Pharmaceutical University, No. 639 Longmian Avenue, Jiangning District, Nanjing, Jiangsu 211198, China; School of International Business, China Pharmaceutical University, No. 639 Longmian Avenue, Jiangning District, Nanjing, Jiangsu 211198, China; Department of Thoracic and Cardiovascular Surgery, Nanjing First Hospital, Nanjing Medical University, Nanjing, Jiangsu, China; Department of Pharmacy, Research Division of Clinical Pharmacology, First Affiliated Hospital of Nanjing Medical University, No. 300 Guangzhou Road, Nanjing, Jiangsu 210029, China; Department of Cardiovascular Medicine, The Affiliated Aoyang Hospital of Jiangsu University, No. 279 Jinggang Road, Zhangjiagang, Suzhou, Jiangsu 215600, China; School of International Business, China Pharmaceutical University, No. 639 Longmian Avenue, Jiangning District, Nanjing, Jiangsu 211198, China

**Keywords:** Leadless pacemaker, TV-VVI, Complications, Mortality, Outcomes, Readmission

## Abstract

**Aims:**

While transvenous pacemakers (TV-VVI) are standard for bradyarrhythmia, lead- and pocket-related complications remain concerns. Leadless pacemakers (LPMs) may reduce these risks. However, direct comparisons between LPMs and single-chamber TV pacemakers are limited. This study aimed to compare clinically meaningful outcomes between LPM and TV-VVI using real-world data.

**Methods and results:**

Using the National Readmissions Database (NRD), we analysed demographics, readmission rates, and 30-day outcomes of patients aged ≥65 years who underwent LPM or TV-VVI implantation between 2016 and 2022. Admissions were identified via ICD-10 codes. Outcomes were assessed in the propensity score-matched population (10 594 patients per group) through multivariable logistic regression after 1:1 high-dimensional propensity score matching (caliper 0.1 SD) to adjust for confounding. Among 49 852 patients, 44.8% received LPM. Median age was 84 vs. 81 years in TV-VVI and LPM groups; 46.2% were female. TV-VVI patients had significantly higher rates of device-related complications [adjusted OR (aOR): 0.45, 95% CI (0.30–0.65), *P* < 0.001], device revision or replacement [aOR: 0.20, 95% CI (0.11–0.36), *P* < 0.001], implant-related complications [aOR: 0.58, 95% CI (0.34–0.97), *P* = 0.040]. Crude rates of arteriovenous fistula, pseudoaneurysm, and pericardial complications were higher in LPM, but adjusted differences were non-significant. Thirty-day readmission rates were similar between LPM and TV-VVI groups at 15.5% and 15.9%, respectively. Mortality and prolonged length of hospital stay also showed no significant differences.

**Conclusion:**

Nationally representative data indicate that LPM implantation is associated with fewer device-related complications compared to TV-VVI, though further studies are needed to evaluate long-term outcomes.

## Graphical Abstract

**Graphical Abstract:**
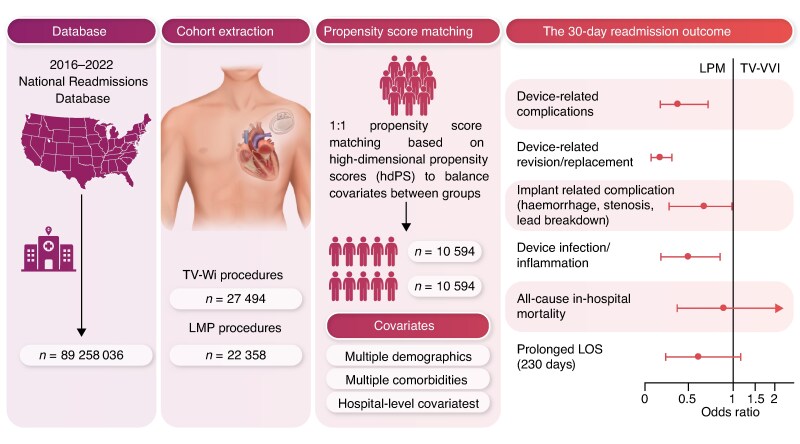

What’s new?

- Leadless pacemaker (LPM) implantation has increased annually, surpassing traditional single-chamber transvenous pacemakers (TV-VVI) in volume since 2020, while TV-VVI implantation has declined overall.
- LPM implantation was associated with fewer device-related complications, including lower rates of device revision or replacement, implant-related complications and device infection.
- No significant differences in 30-day readmission rates were observed between the LPM and TV-VVI groups.
- No significant differences in all-cause in-hospital mortality or prolonged length of stay (≥30 days) were observed between the LPM and TV-VVI groups within 30 days of readmission.

## Introduction

With the accelerating process of global population aging, the demand for pacemaker implantation has been steadily increasing. According to the 2021 European Society of Cardiology (ESC) Guidelines for Cardiac Pacing and Resynchronization Therapy, ∼1 million cardiac devices are implanted globally each year, and this trend continues to rise.^1^ Pacemakers are essential medical devices for the treatment of bradyarrhythmia, such as atrioventricular block and sick sinus syndrome.^1–3^

Although the traditional single-chamber transvenous pacemakers (TV-VVI) have been widely used and offer significant clinical benefits, the presence of leads and the subcutaneous pulse generator pocket may cause device-related complications.^4^  ^,5^

In recent years, leadless pacemakers (LPM) have emerged as a novel technology. LPM avoid the need for leads and the pocket structure typically required by traditional pacemakers. The first single-chamber right ventricular LPM received CE certification in 2015 and FDA approval in 2016. Early studies demonstrated a high implantation success rate of up to 99.6% and a primary complication rate of 1.51%.^6^ As outlined in the 2021 ESC guidelines, LPM are considered a suitable alternative for high-risk patients, including those with a history of pocket infections, those undergoing haemodialysis, and those with venous access obstruction that precludes the implantation of traditional transvenous pacemakers (TVPs).^1^

However, randomized controlled trials comparing LPM and TV-VVI are limited, and real-world evidence remains scarce. Registry data in haemodialysis patients suggest that LPM are associated with lower device-related infections and improved survival compared with single-chamber transvenous devices; however, these findings are restricted to a high-risk population and may not generalize to older adults with broader comorbidity profiles.^7^

Hospital readmission is a widely accepted indicator of healthcare quality and short-term patient outcomes, particularly in cardiovascular care.^8^ Older adults receiving pacemakers often have multiple comorbidities and are prone to early rehospitalizations.^9^ Therefore, 30-day readmission is a clinically meaningful outcome to assess short-term differences in outcomes between leadless and transvenous pacing in real-world practice. Therefore, we aim to evaluate clinical outcomes, including 30-day readmission, between LPM and TV-VVI in this large real-world retrospective cohort study based on the National Readmissions Database (NRD). A high-dimensional propensity score (HDPS) matching approach was applied to balance baseline characteristics and reduce confounding.

## Methods

### Data sources

This study utilized data from the NRD spanning 2016–22. The NRD is part of the Healthcare Cost and Utilization Project (HCUP), supported by the Agency for Healthcare Research and Quality (AHRQ) and contains patient-level linkage variables that enable tracking of readmissions across hospitals within a calendar year. It includes nationally representative in-patient discharge records from multiple U.S. states, covering ∼35 million weighted discharges annually (excluding rehabilitation and long-term acute care facilities). All data are de-identified, and thus, Institutional Review Board approval was not required. Data for the study were systematically obtained using the International Classification of Diseases, Tenth Revision, Clinical Modification and Procedure Coding System (ICD-10-CM/PCS) for the identification of diagnoses and procedures, respectively. The database also includes key clinical variables, such as patient demographics, in-hospital mortality, and length of stay.

### Sample extraction

We queried the NRD from 2016 to 2022 to include patients aged ≥65 years with an admission diagnosis of sick sinus syndrome, second-degree atrioventricular block, or third-degree atrioventricular block, who underwent either a LPM or TV-VVI implantation during hospitalization. LPM implantation was identified using the ICD-10-PCS code (02HK3NZ), which specifically denotes an entirely intracardiac device without transvenous leads. TV-VVI was defined by the presence of both single-chamber pacemaker codes (0JH604Z, 0JH605Z) and right ventricular lead insertion codes (02HK3JZ, 02HK4JZ, 02HK3MZ, 02HK4MZ). Internal checks confirmed that no LPM-coded patients had concurrent lead insertion codes, ensuring accurate classification.

Patients were excluded if they had a prior history of cardiovascular implantable electronic devices (CIEDs), were younger than 65 years, or had missing in-hospital mortality information. The hospitalization during which the pacemaker was implanted was defined as the ‘index admission’, and any readmission occurring within a specified readmission period after discharge from the index admission was defined as the corresponding ‘readmission’ (e.g. 30-day readmission). If multiple readmissions occurred within the same readmission window, only the first readmission was included. To ensure the accuracy of readmission rate estimation across different readmission periods, patients with insufficient observation window were excluded, specifically, patients enrolled in December were excluded from the 30-day readmission analysis. We additionally analysed the causes of 30-day readmissions, which were classified based on the primary diagnosis ICD-10-CM code at readmission. Causes were categorized as cardiac or non-cardiac, and further stratified as elective or non-elective.

### Statistical analysis

To control for potential confounders, a range of patient-level and hospital-level covariates were included in this study. Patient-level covariates included: age, sex, insurance type, median household income for the patient's zip code, Charlson comorbidity index (CCI) score, aortic aneurysm, atrial fibrillation, cardiac aneurysm, percutaneous coronary intervention (PCI), ischaemic heart disease (IHD), myocardial infarction (MI), coronary artery bypass grafting (CABG), non-ST elevation myocardial infarction (NSTEMI), ST elevation myocardial infarction (STEMI), dementia, ischaemic stroke, haemorrhagic stroke, transient ischaemic attack (TIA), dyslipidaemia, hypertension, diabetes, obesity, coronary artery disease (CAD), peripheral artery disease (PAD), chronic kidney disease (CKD), end-stage renal disease (ESRD), chronic obstructive pulmonary disease (COPD), congestive heart failure (CHF), cardiogenic shock, anaemia, thrombocytopenia, chronic liver disease, tobacco use, alcohol use, substance abuse, history of malignancy, and venous thromboembolism. Hospital-level covariates included hospital bed size and hospital location/teaching status.

Categorical variables were presented as frequencies and percentages, while continuous variables were reported using medians and inter-quartile ranges (IQRs).

To further control for high-dimensional confounders, we employed the HDPS method. HDPS uses large-scale diagnostic and procedural coding information, combining the frequency of variables and their association strength with the study outcome.^10^ From the patient's diagnostic and procedural codes, the 100 most relevant variables were selected and included in the propensity score model, alongside the pre-specified clinical covariates.^11,12^

Subsequently, propensity score matching (PSM) was performed using a 1:1 nearest-neighbour matching method without replacement and employing a greedy algorithm. A caliper width of 0.1 standard deviations (SD) was used to construct a well-matched propensity-matched cohort, ensuring independence of matched pairs to improve the validity of statistical inference and reduce potential bias, even though this reduced the sample size. The balance of baseline characteristics was primarily assessed using standardized mean differences (SMD), with an SMD < 0.1 indicating negligible differences. Before matching, crude odds ratios (OR) were estimated using a univariate logistic regression model. After matching, multivariate logistic regression was performed on the matched cohort to estimate the adjusted odds ratios (aOR). The aOR model included covariates that remained imbalanced after matching (SMD > 0.1) as well as covariates with SMD close to 0.1 that were considered clinically important.

To supplement the observation of longer-term clinical outcomes and enhance the robustness of our findings, sensitivity analyses were conducted to assess readmission outcomes at 60, 90, and 180 days.

All analyses were conducted using SAS version 9.4 (SAS Institute Inc., Cary, NC, USA) and R version 4.3.1 (R Foundation for Statistical Computing, Vienna, Austria). Two-sided statistical tests with an α level of <0.05 were considered statistically significant.

## Results

### Baseline characteristic and readmission rate

**Figure 1:**
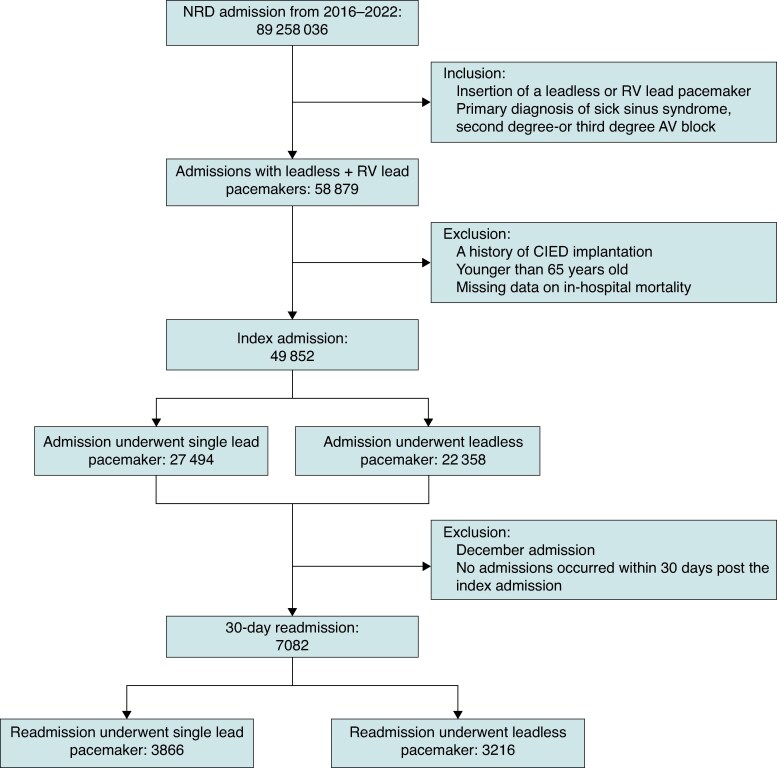
Consort diagram showing the derivation of our study sample. RV, right ventricle; CIED, cardiovascular implantable electronic device.

**Figure 2:**
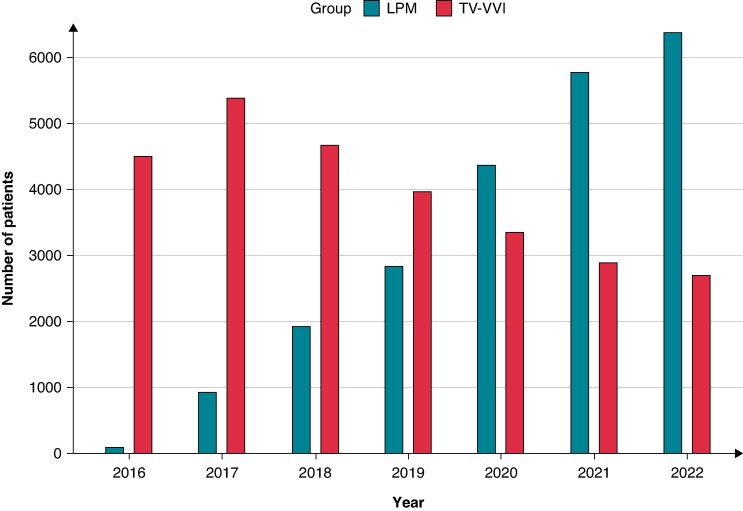
Temporal trends of LPM or traditional TVP. LPM, leadless pacemaker; TV-VVI, transvenous single-chamber ventricular pacemaker.

Our cohort included 49 852 patients who underwent pacemaker implantation during the study period. Of these, 27 494 (55.2%) received TV-VVI, and 22 358 (44.8%) received LPM (*Figure 1*). Between 2016 and 2022, the number of LPM implantations increased markedly from 98 to 6388. LPM implantations surpassed TV-VVI in volume beginning in 2020 and continued to exceed them thereafter (*Figure 2*). Overall, the majority of patients were aged ≥80 years (67.8% in the TV-VVI group and 56.8% in the LPM group), and 46.2% were female. Most implantations were performed at large, urban teaching hospitals. The most prevalent comorbidities were atrial fibrillation (74.7%), hypertension (65.2%), hyperlipidaemia (60.7%), and heart failure (54.4%) (*Table 1*). Compared with the LPM group, the TV-VVI group had a higher prevalence of atrial fibrillation (85.7% vs. 61.1%), whereas the LPM group had a higher prevalence of ESKD (10.5% vs. 2.7%). In the overall cohort, the 30-day readmission rates were 15.9% in the LPM group and 15.3% in the TV-VVI group, no significant difference was observed [OR: 1.05, 95% CI (0.99–1.10), *P* = 0.079] (see Supplementary material online, *Figure S1* and Supplementary material online, *Table S5*). Among 30-day readmissions, 40.8% were due to cardiac causes and 59.2% to non-cardiac causes. The majority of admissions were non-elective, accounting for 88.1% (see Supplementary material online, *Table S1*).

**Table 1:** Baseline characteristics of pacemaker implantation patients

Variable	Overall	Before propensity matching			After propensity matching		
		Treatment		|SMD|	Treatment		|SMD|
		TV-VVI	Leadless		TV-VVI	Leadless	
Population	49 852	27 494	22 358		10 594	10 594	
Age	83 [76, 88]	84 [78, 89]	81 [74, 87]	0.264	82 [76, 88]	82 [76, 88]	0.022
Age group (%)							
65–69	3847 (7.7)	1594 (5.8)	2253 (10.1)	0.251	809 (7.6)	844 (8.0)	0.028
70–74	6192 (12.4)	2840 (10.3)	3352 (15.0)		1307 (12.3)	1392 (13.1)	
75–79	8485 (17.0)	4426 (16.1)	4059 (18.2)		1873 (17.7)	1849 (17.5)	
80+	31 328 (62.8)	18 634 (67.8)	12 694 (56.8)		6605 (62.3)	6509 (61.4)	
Female sex (%)	23 008 (46.2)	12 695 (46.2)	10 313 (46.1)	0.001	4903 (46.3)	4888 (46.1)	0.003
Insurance type (%)							
Medicare	45 956 (92.2)	25 510 (92.8)	20 446 (91.4)	0.080	9724 (91.8)	9663 (91.2)	0.030
Medicaid	555 (1.1)	246 (0.9)	309 (1.4)		126 (1.2)	136 (1.3)	
Private Insurance	2132 (4.3)	1185 (4.3)	947 (4.2)		460 (4.3)	486 (4.6)	
Self-pay	136 (0.3)	61 (0.2)	75 (0.3)		31 (0.3)	33 (0.3)	
Other	1034 (2.1)	478 (1.7)	556 (2.5)		244 (2.3)	264 (2.4)	
Missing	39 (0.1)	14 (0.1)	25 (0.1)		9 (0.1)	12 (0.1)	
Median household income for the patient zip code, *n* (%)							
Q1: 0–25th percentile	10 835 (21.7)	5934 (21.6)	4901 (21.9)	0.080	2280 (21.5)	2259 (21.3)	0.012
Q2: 26th−50th percentile	13 055 (26.2)	7577 (27.6)	5478 (24.5)		2804 (26.5)	2815 (26.6)	
Q3: 51st–75th percentile	12 861 (25.8)	7047 (25.6)	5814 (26.0)		2741 (25.9)	2747 (25.9)	
Q4: 76th–100th percentile	12 564 (25.2)	6622 (24.1)	5942 (26.6)		2667 (25.2)	2659 (25.1)	
Missing	537 (1.1)	314 (1.1)	223 (1.0)		102 (1.0)	114 (1.1)	
CCI (%)							
0–5	9494 (19.0)	5110 (18.6)	4384 (19.6)	0.081	2016 (19.0)	2004 (18.9)	0.008
6–7	17 314 (34.7)	10 021 (36.4)	7293 (32.6)		3607 (34.0)	3575 (33.7)	
8+	23 044 (46.2)	12 363 (45.0)	10 681 (47.8)		4971 (46.9)	5015 (47.3)	
Bed size (%)							
Small	5541 (11.1)	3714 (13.5)	1827 (8.2)	0.276	995 (9.4)	1106 (10.4)	0.050
Medium	13 450 (27.0)	8354 (30.4)	5096 (22.8)		2836 (26.8)	2640 (24.9)	
Large	30 861 (61.9)	15 426 (56.1)	15 435 (69.0)		6763 (63.8)	6848 (64.6)	
Location/teaching status (%)							
Rural	10 607 (21.3)	6702 (24.4)	3905 (17.5)	0.292	2180 (20.6)	1797 (17.0)	0.227
Urban teaching	37 619 (75.5)	19 434 (70.7)	18 185 (81.3)		7917 (74.7)	8657 (81.7)	
Urban non-teaching	1626 (3.3)	1358 (4.9)	268 (1.2)		497 (4.7)	140 (1.3)	
Years (%)							
2016	4612 (9.3)	4514 (16.4)	98 (0.4)	1.084	416 (3.9)	94 (0.9)	0.311
2017	6320 (12.7)	5390 (19.6)	930 (4.2)		956 (9.0)	808 (7.6)	
2018	6603 (13.2)	4677 (17.0)	1926 (8.6)		1377 (13.0)	1512 (14.3)	
2019	6807 (13.7)	3962 (14.4)	2845 (12.7)		1680 (15.9)	1979 (18.7)	
2020	7746 (15.5)	3359 (12.2)	4387 (19.6)		1816 (17.1)	2348 (22.2)	
2021	8669 (17.4)	2885 (10.5)	5784 (25.9)		2017 (19.0)	2320 (21.9)	
2022	9095 (18.2)	2707 (9.8)	6388 (28.6)		2332 (22.0)	1533 (14.5)	
Comorbidities (%)							
Aortic Aneurysm	1385 (2.8)	778 (2.8)	607 (2.7)	0.007	300 (2.8)	306 (2.9)	0.003
Atrial Fibrillation	37 219 (74.7)	23 560 (85.7)	13 659 (61.1)	0.580	8015 (75.7)	7785 (73.5)	0.050
Cardiac Aneurysm	49 (0.1)	21 (0.1)	28 (0.1)	0.015	12 (0.1)	9 (0.1)	0.009
PCI	5220 (10.5)	2841 (10.3)	2379 (10.6)	0.010	1113 (10.5)	1117 (10.5)	0.001
IHD	22 208 (44.5)	12 239 (44.5)	9969 (44.6)	0.001	4726 (44.6)	4749 (44.8)	0.004
MI	4471 (9.0)	2458 (8.9)	2013 (9.0)	0.002	957 (9.0)	955 (9.0)	0.001
CABG	5415 (10.9)	3138 (11.4)	2277 (10.2)	0.040	1147 (10.8)	1105 (10.4)	0.013
NSTEMI	1836 (3.7)	941 (3.4)	895 (4.0)	0.031	413 (3.9)	424 (4.0)	0.005
STEMI	391 (0.8)	170 (0.6)	221 (1.0)	0.041	105 (1.0)	104 (1.0)	0.001
Dementia	8310 (16.7)	4643 (16.9)	3667 (16.4)	0.013	1825 (17.2)	1837 (17.3)	0.003
Stroke, Ischaemic	2861 (5.7)	1545 (5.6)	1316 (5.9)	0.011	632 (6.0)	598 (5.6)	0.014
Stroke, Haemorrhagic	365 (0.7)	174 (0.6)	191 (0.9)	0.026	83 (0.8)	93 (0.9)	0.010
TIA	398 (0.8)	270 (1.0)	128 (0.6)	0.047	66 (0.6)	76 (0.7)	0.012
Dyslipidaemia	30 270 (60.7)	16 404 (59.7)	13 866 (62.0)	0.048	6466 (61.0)	6471 (61.1)	0.001
Hypertension	32 502 (65.2)	18 322 (66.6)	14 180 (63.4)	0.068	6785 (64.0)	6735 (63.6)	0.010
Diabetes	18 415 (36.9)	9504 (34.6)	8911 (39.9)	0.110	3909 (36.9)	3991 (37.7)	0.016
Obesity	7650 (15.3)	4188 (15.2)	3462 (15.5)	0.007	1622 (15.3)	1654 (15.6)	0.008
CAD	22 168 (44.5)	12 217 (44.4)	9951 (44.5)	0.001	4719 (44.5)	4744 (44.8)	0.005
PAD	4581 (9.2)	2648 (9.6)	1933 (8.6)	0.034	972 (9.2)	967 (9.1)	0.002
CKD	19 210 (38.5)	10 108 (36.8)	9102 (40.7)	0.081	4129 (39.0)	4128 (39.0)	<0.001
ESRD	3108 (6.2)	755 (2.7)	2353 (10.5)	0.316	550 (5.2)	658 (6.2)	0.044
COPD	11 974 (24.0)	6756 (24.6)	5218 (23.3)	0.029	2561 (24.2)	2553 (24.1)	0.002
CHF	27 095 (54.4)	15 517 (56.4)	11 578 (51.8)	0.093	5803 (54.8)	5781 (54.6)	0.004
Cardiogenic Shock	2580 (5.2)	1037 (3.8)	1543 (6.9)	0.140	630 (5.9)	625 (5.9)	0.002
Anaemia	3425 (6.9)	1787 (6.5)	1638 (7.3)	0.033	774 (7.3)	751 (7.1)	0.008
Thrombocytopenia	4676 (9.4)	2446 (8.9)	2230 (10.0)	0.037	993 (9.4)	989 (9.3)	0.001
Chronic Liver Disease	1561 (3.1)	731 (2.7)	830 (3.7)	0.060	368 (3.5)	377 (3.6)	0.005
Tobacco Use	16 027 (32.1)	8858 (32.2)	7169 (32.1)	0.003	3398 (32.1)	3402 (32.1)	0.001
Alcohol Use	1035 (2.1)	548 (2.0)	487 (2.2)	0.013	239 (2.3)	221 (2.1)	0.012
Substance Abuse	2688 (5.4)	1364 (5.0)	1324 (5.9)	0.042	594 (5.6)	611 (5.8)	0.007
History of Malignancy	8274 (16.6)	4506 (16.4)	3768 (16.9)	0.012	1748 (16.5)	1765 (16.7)	0.004
Venous Thromboembolism	1967 (3.9)	802 (2.9)	1165 (5.2)	0.116	441 (4.2)	470 (4.4)	0.013

CCI, Charlson comorbidity index; PCI, percutaneous coronary intervention; IHD, ischaemic heart disease; MI, myocardial infarction; CABG, coronary artery bypass grafting; NSTEMI, non-ST elevation myocardial infarction; STEMI, ST elevation myocardial infarction; TIA, transient ischaemic attack; CAD, coronary artery disease; PAD, peripheral artery disease; CKD, chronic kidney disease; ESRD, end-stage renal disease; COPD, chronic obstructive pulmonary disease; CHF, congestive heart failure.

### In-patient mortality, length of stay, and complications

**Figure 3:**
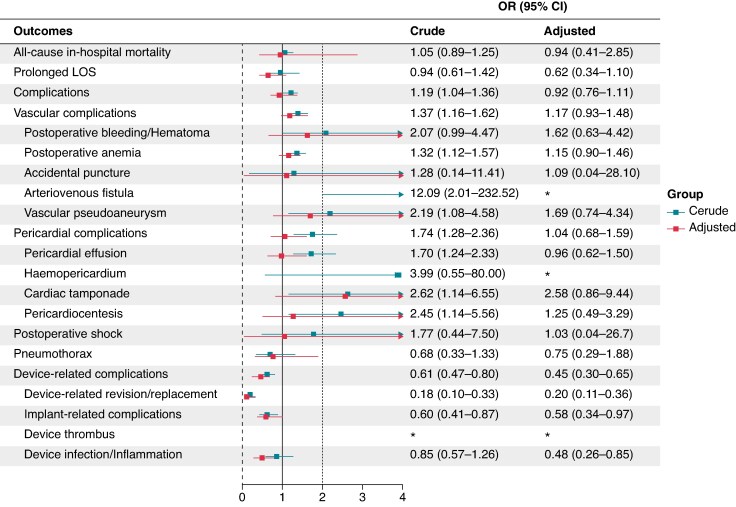
Breakdown of primary outcomes and logistic regression before and after PSM among patients with 30-day rehospitalization. OR compares LPMs to TV-VVI pacemakers; OR < 1 indicates lower risk in the leadless group.

After 1:1 HDPS matching, a total of 21 188 hospitalizations were included, with 10 594 patients in each group. All subsequent outcome analyses, including in-hospital mortality, length of stay (LOS), and complications, were conducted in the matched population. In this cohort, all-cause in-hospital mortality during 30-day readmission was 9.3% in the LPM group and 10.3% in the TV-VVI group [aOR: 0.94, 95% CI (0.41–2.85), *P* = 0.881], with no statistically significant difference. Similarly, prolonged length of stay (≥30 days) occurred in 1.3% of the LPM group and 2.0% of the TV-VVI group [aOR: 0.62, 95% CI (0.34–1.10), *P* = 0.107], also without a significant difference (*Table 2* and *Figure 3*).

**Table 2:** Breakdown of primary outcomes and logistic regression analysis before and after PSM among patients with 30-day rehospitalization

Primary outcome	Before propensity matching			After propensity matching			Crude OR^a^ (95% CI)	*P*-value	Adjusted OR^a^ (95% CI)	*P*-value
	Overall *n* = 7082 (%)	Leadless *n* = 3216 (%)	TV-VVI *n* = 3866 (%)	Overall *n* = 3046 (%)	Leadless *n* = 1519 (%)	TV-VVI *n* = 1527 (%)				
All-cause in-hospital mortality	643 (9.1)	299 (9.3)	344 (8.9)	300 (9.8)	142 (9.3)	158 (10.3)	1.05 (0.89–1.25)	0.551	0.94 (0.41–2.85)	0.881
Prolonged LOS^a^ (≥30 days)	101 (1.4)	45 (1.4)	56 (1.4)	49 (1.6)	19 (1.3)	30 (2.0)	0.94 (0.61–1.42)	0.761	0.62 (0.34–1.10)	0.107
Complications	1090 (15.4)	539 (16.8)	551 (14.3)	494 (16.2)	238 (15.7)	256 (16.8)	1.19 (1.04–1.36)	0.014	0.92 (0.76–1.11)	0.376
Vascular complications	709 (10.0)	373 (11.6)	336 (8.7)	328 (10.8)	176 (11.6)	152 (10.0)	1.37 (1.16–1.62)	<0.001	1.17 (0.93–1.48)	0.169
Post-operative bleeding/Hematoma	31 (0.4)	19 (0.6)	12 (0.3)	18 (0.6)	11 (0.7)	<11^b^	2.07 (0.99–4.47)	0.055	1.62 (0.63–4.42)	0.324
Post-operative anaemia	666 (9.4)	346 (10.8)	320 (8.3)	301 (9.9)	160 (10.5)	141 (9.2)	1.32 (1.12–1.57)	<0.001	1.15 (0.90–1.46)	0.266
Accidental puncture	<11^b^	<11^b^	<11^b^	<11^b^	<11^b^	<11^b^	1.28 (0.14–11.41)	0.810	1.09 (0.04–28.10)	0.950
Arteriovenous fistula	<11^b^	<11^b^	<11^b^	<11^b^	<11^b^	0	12.09 (2.01–232.52)	0.023	*	0.992
Vascular pseudoaneurysm	35 (0.5)	22 (0.7)	13 (0.3)	24 (0.8)	15 (1.0)	<11^b^	2.19 (1.08–4.58)	0.032	1.69 (0.74–4.34)	0.216
Pericardial complications	192 (2.7)	114 (3.5)	78 (2.0)	89 (2.9)	45 (3.0)	44 (2.9)	1.74 (1.28–2.36)	<0.001	1.04 (0.68–1.59)	0.861
Pericardial effusion	183 (2.6)	108 (3.4)	75 (1.9)	82 (2.7)	40 (2.6)	42 (2.8)	1.70 (1.24–2.33)	<0.001	0.96 (0.62–1.50)	0.869
Haemopericardium	<11^b^	<11^b^	<11^b^	<11^b^	<11^b^	0	3.99 (0.55–80.00)	0.226	*	0.997
Cardiac tamponade	27 (0.4)	19 (0.6)	<11^b^	14 (0.5)	<11^b^	<11^b^	2.62 (1.14–6.55)	0.028	2.58 (0.86–9.44)	0.111
Pericardiocentesis	31 (0.4)	21 (0.7)	<11^b^	18 (0.6)	<11^b^	<11^b^	2.45 (1.14–5.56)	0.025	1.25 (0.49–3.29)	0.636
Post-operative shock	<11^b^	<11^b^	<11^b^	<11^b^	<11^b^	<11^b^	1.77(0.44–7.50)	0.414	1.03 (0.04–26.7)	0.981
Pneumothorax	39 (0.6)	14 (0.4)	25 (0.6)	19 (0.6)	<11^b^	11 (0.7)	0.68 (0.33–1.33)	0.267	0.75 (0.29–1.88)	0.544
Device-related complications	276 (3.9)	101 (3.1)	175 (4.5)	124 (4.1)	39 (2.6)	85 (5.6)	0.61 (0.47–0.80)	<0.001	0.45 (0.30–0.65)	<0.001
Device-related revision/replacement	96 (1.4)	13 (0.4)	83 (2.1)	48 (1.6)	<11^b^	40 (2.6)	0.18 (0.10–0.33)	<0.001	0.20 (0.11–0.36)	<0.001
Implant related complication (Hemorrhage, stenosis, lead breakdown)	141 (2.0)	53 (1.6)	88 (2.3)	62 (2.0)	23 (1.5)	39 (2.6)	0.60 (0.41–0.87)	0.007	0.58 (0.34–0.97)	0.040
Device thrombus	<11^b^	0	<11^b^	<11^b^	0	<11^b^	*	0.996	*	0.998
Device infection/Inflammation	112 (1.6)	48 (1.5)	64 (1.7)	52 (1.7)	17 (1.1)	35 (2.3)	0.85 (0.57–1.26)	0.417	0.48 (0.26–0.85)	0.014

^a^Crude OR, odds ratio before propensity score matching; Adjusted OR: odds ratio after propensity score matching; CI, confidence interval; PSM, propensity score matching; LOS, length of stay; TV-VVI, transvenous ventricular-inhibited pacing.

^b^Per HCUP policy, counts <11 are suppressed to protect privacy.

As shown in *Table 2*, the incidence of arteriovenous fistula was 0.2% in the LPM group and <0.1% in the TV-VVI group; adjusted analysis was not feasible due to the very low number of events. Vascular pseudoaneurysm occurred more frequently in the LPM group (1.0% vs. 0.6%), with a non-significant trend after adjustment [aOR: 1.69; 95% CI (0.74–4.34); *P* = 0.216]. Pericardial complications occurred in 3.0% of LPM patients and 2.9% of TV-VVI patients after adjustment [aOR: 1.04; 95% CI (0.68–1.59); *P* = 0.861], representing a non-significant trend. In contrast, device-related complications were significantly lower in the LPM group (2.6% vs. 5.6%) [aOR: 0.45; 95% CI (0.30–0.65); *P* < 0.001], as were device infections or inflammations (1.1% vs. 2.3%) [aOR: 0.48; 95% CI (0.26–0.85); *P* = 0.014] (*Table 2* and *Figure 3*).

### Sensitivity analyses for longer-term readmission

The sensitivity analyses showed that the readmission rates at 60, 90, and 180 days were similar between groups and the trends for in-hospital mortality and major complications were consistent with the primary 30-day results. Detailed numerical results are provided in the Supplementary Materials.

## Discussion

This real-world, population-based study included 49 852 readmitted patients, of whom 27 494 (55.2%) received TV-VVI and 22 358 (44.8%) received LPM. We analysed one of the largest contemporary retrospective cohorts comparing LPM and TV-VVI implantations and identified several key findings: (i) LPM adoption increased steadily over time, surpassing TV-VVI volumes in 2020, while TV-VVI use declined; (ii) Despite this trend, TV-VVI recipients experienced higher rates of device-related complications, including revisions or replacements, implant-site issues, and device-associated infections or inflammations; (iii) In contrast, 30-day readmission rates, all-cause in-hospital mortality, and prolonged length of stay (≥30 days) after readmission were similar between groups; (iv) Although unadjusted data indicated more frequent arteriovenous fistulas, vascular pseudoaneurysms, and pericardial complications among LPM patients during readmission, these represent non-significant trends after multivariable adjustment.

We observed 30-day readmission rates of 15.9% and 15.3% in the LPM and TV-VVI groups, respectively. A previous study based on the NRD reported a 30-day readmission rate of ∼13% following transvenous permanent pacemaker implantation,^13^ while another study focusing on LPMs demonstrated a higher all-cause readmission rate of 17.9%,^14^ aligning closely with our LPM findings. Moreover, we found no statistically significant difference in readmission rates between the LPM and TV-VVI groups, consistent with Mararenko *et al*.^15^

Regarding short-term mortality among readmitted patients, unadjusted all-cause mortality was initially lower in the TV-VVI group. However, after adjustment for confounders, the TV-VVI group showed a slightly higher mortality rate (10.3% vs. 9.3%), representing a non-significant trend. This suggests that baseline illness severity, rather than device type, may have driven mortality. Prior NRD analyses noted that LPM recipients often have greater comorbidity burdens, including end-stage renal disease and chronic haemodialysis, both of which are recognized as primary indications for LPM use.^14^ Moreover, a meta-analysis involving 2496 patients found no significant difference in all-cause mortality between the groups [RR: 0.45, 95% CI (0.15–1.35), *P* = 0.160].^16^ The current literature on both 30-day and long-term mortality outcomes is generally consistent with our findings.^17,18^ Differences compared with previous NRD studies reporting higher short-term mortality in LPM recipients are likely due to differences in study period and statistical methods. Our analysis included 2016–22, capturing the period of rapidly increasing LPM use, while other studies focused on earlier years.^19^ Variations in matching criteria and statistical approaches may also contribute to these discrepancies.

Concerning procedural outcomes, several studies have compared LPMs and transvenous systems.^20–24^ Breeman *et al*. reported that although LPM recipients may experience certain acute, procedure-related complications in the early post-operative phase, their long-term complication rates remain low, and pacing performance is maintained over a 5-year follow-up period.^22^ A Danish cohort study involving 5918 patients found that 9.5% experienced at least one complication during follow-up.^25^ Other studies have suggested that LPMs are associated with fewer complications compared to conventional transvenous systems,^23^ which aligns with our results, though adjusted differences were not statistically significant.

For vascular complications, arteriovenous fistula (0.4%) and pseudoaneurysm (1.0%) were more frequent in the LPM group, representing non-significant trends. This may be closely linked to the unique implantation approach and procedural characteristics. LPM are typically delivered via the femoral vein using an introducer sheath up to 23 French in diameter, which is significantly larger than the sheaths used for conventional transvenous systems.^26–28^ This may increase the risk of vascular injury at the access site and contribute to a higher rate of post-procedural complications. These non-significant trends should be interpreted cautiously and do not imply definitive clinical differences between the groups. Due to limited follow-up duration and small event counts, these results may not reflect potential differences in longer-term follow-up. Roberts *et al*. reported that within 30 days after LPM implantation, 13 major complications occurred in 12 patients, with an overall incidence of 1.51%, including arteriovenous fistula (0.13%) and vascular pseudoaneurysm (0.13%).^6^ In a prospective, single-arm clinical study that enrolled 744 patients, 725 patients underwent attempted device implantation, and 719 were successfully implanted. A total of 28 major complications were reported, including 11 cases of cardiac injury and 5 cases of groin puncture site complications.^29^ The complication rates reported in this study were comparable to those observed in our analysis.

Regarding cardiac injury, our findings showed that prior to adjustment, the LPM group had higher rates of pericardial effusion, cardiac tamponade, and pericardiocentesis; however, after adjusting for confounding factors, there was no statistically significant difference between the groups. This may be attributed to the device’s structure. Although LPM is compact, it is encased in a hard metal shell, with a nitinol anchor hook at the front that directly attaches to the right ventricular wall. If the fixation is too deep or misaligned, it may puncture or tear the myocardium, leading to complications such as pericardial effusion, pericarditis, or even cardiac tamponade. Previous reports on the acute and six-month outcomes of the Micra CED study found a higher incidence of pericardial effusion and/or perforation in patients implanted with leadless VVI pacemakers.^30^ In a retrospective study, Sattar *et al*. analysed 204 patients with LPM and found that the risk of pericardial effusion was approximately twice that of traditional pacemakers, although this difference did not reach statistical significance, representing a non-significant trend.^31^ Some studies have also found a higher incidence of pericarditis and pericardial effusion, which are similar to our results.^17,24,32^

Regarding device-related complications, our study found that LPM recipients experienced lower rates of device revisions, implant-site issues, and device-related infections, whereas TV-VVI recipients experienced more complications, most of which were lead- or pocket-related. Even if not immediately fatal, these lead- or pocket-related complications substantially increase healthcare resource use, hospitalization costs, and the likelihood of repeat interventions.^33,34^ Long-term evidence further indicates that device-related infections significantly elevate mortality risk: cardiac device infection after transvenous lead extraction was associated with higher 1-year and 10-year mortality,^35^ and in a real-world CIED cohort, infection was associated with a 12-month adjusted hazard ratio for death of 2.73 (95% CI 2.10–3.54) compared with uninfected patients.^36^ Collectively, these findings underscore the clinical importance of preventing TV-VVI complications, both to reduce healthcare burden and to avoid potentially life-threatening outcomes. The lower complication rate in LPM recipients can be attributed to design features that avoid the need for a subcutaneous pocket and transvenous leads.^24,30^ The LPM is compact, lightweight, and precisely engineered, incorporating high-density batteries, low-power electronics, catheter-based delivery systems, and materials like nitinol.^15,37,38^ As a single-chamber device, it is implanted directly into the right ventricular wall via the femoral vein, eliminating the need for a subcutaneous pocket or transvenous leads.^26,28,39^ This design minimizes typical complications associated with traditional pacemakers, such as infection, lead dislodgement, and mechanical failure, thereby enhancing safety, particularly in patients at elevated risk for conventional systems. Cantillon *et al.* reported that within both the short- to mid-term period (≤1 month) and the mid-term period (>1–18 months), leadless cardiac pacemaker (LCP) recipients did not experience TVP-related complications such as lead failure, pocket complications, or device-related infections.^24,26,39^ These data support that LCP significantly reduce the risk of device-related complications and infections.

### Limitations

Several limitations should be considered when interpreting the findings of this study. First, the analysis was based on a large administrative database in which comorbidities and complications were identified through ICD codes. While these coding algorithms have been widely validated in prior research, their accuracy in routine clinical settings remains variable. Nonetheless, any misclassification is likely to be non-differential and would therefore bias the results towards the null. Second, the retrospective observational design precludes definitive causal inference and may be subject to residual confounding, despite multivariable adjustment. A major constraint of the National Readmissions Database is the absence of granular clinical data such as medication use, laboratory parameters, hemodynamic status, or pacing indications. Consequently, we were unable to determine the precise causes of in-hospital mortality, and deaths occurring outside the hospital were not captured. While causality cannot be firmly established, it is likely that bradycardia-related deaths reflect underlying comorbid conditions rather than device-related failure. Third, comparisons with other studies should be interpreted cautiously due to potential differences in outcome definitions and populations. The lack of longitudinal linkage across states or calendar years also limits the ability to capture readmissions comprehensively, potentially underestimating the true readmission burden. Moreover, the dataset does not include device-specific characteristics such as pacing thresholds, battery longevity, or implantation techniques, limiting our ability to explore potential mechanisms underlying the observed outcome differences. Finally, the present study focused exclusively on single-chamber LPMs; recent reports on dual-chamber–capable LPMs suggest differing complication and survival profiles, highlighting that our findings may not be directly extrapolated to these newer devices.^40^

## Conclusions

Overall, LPM demonstrated significantly lower device-related complications, driven by reduced infections and lead revisions, despite comparable in the short-term readmission rates and in-hospital mortality between LPM and TV-VVI. Large-scale, prospective, randomized controlled trials are needed to further elucidate the long-term risks and clinical benefits of LPMs compared to traditional transvenous systems. These findings pertain only to single-chamber LPMs and may not be generalizable to dual-chamber leadless systems.

## Supplementary Material

euaf268_Supplementary_Data

## Data Availability

The data underlying this article are available in the National Readmissions Database at https://www.hcup-us.ahrq.gov/ and can be accessed by purchasing them directly from the HCUP website.
