# Supplementary material for: Comparison of readmission outcomes and complications between leadless and traditional transvenous pacemakers in older adults: a nationwide readmission analysis of 49852 admission events
Source: Europace. 2025 Oct 29;27(11):euaf268. doi: 10.1093/europace/euaf268 (PMC12596647; doi:10.1093/europace/euaf268)
Supplement: euaf268_Supplementary_Data [file euaf268_supplementary_data.docx]

**Supplementary materials**

**Title:** Comparison of Readmission Outcomes and Complications Between Leadless and Traditional Transvenous Pacemakers in Older Adults: A Nationwide Readmission Analysis of 49852 Admission Events

**List of Figures**

**Figure S1.** Risk of rehospitalization among patients with leadless and TV-VVI pacemakers before and after propensity score matching (PSM).

**Figure S2.** Love plot of covariate balance (|SMD|) before and after PSM.

**Figure S3.** Breakdown of primary outcomes and logistic regression before and after PSM among patients with 60-day rehospitalization.

**Figure S4.** Breakdown of primary outcomes and logistic regression before and after PSM among patients with 90-day rehospitalization.

**Figure S5.** Breakdown of primary outcomes and logistic regression before and after PSM among patients with 180-day rehospitalization.

**List of Tables**

**Table S1.** Causes of 30-day readmission pacemaker implantation patients.

**Table S2**. Breakdown of primary outcomes and logistic regression analysis before and after PSM among patients with 60-day rehospitalization.

**Table S3**. Breakdown of primary outcomes and logistic regression analysis before and after PSM among patients with 90-day rehospitalization.

**Table S4**. Breakdown of primary outcomes and logistic regression analysis before and after PSM among patients with 180-day rehospitalization.

**Table S5.** Comparison of readmission rates before and after PSM.

**Figure S1.** Risk of rehospitalization among patients with leadless and TV-VVI pacemakers before and after propensity score matching (PSM).

**
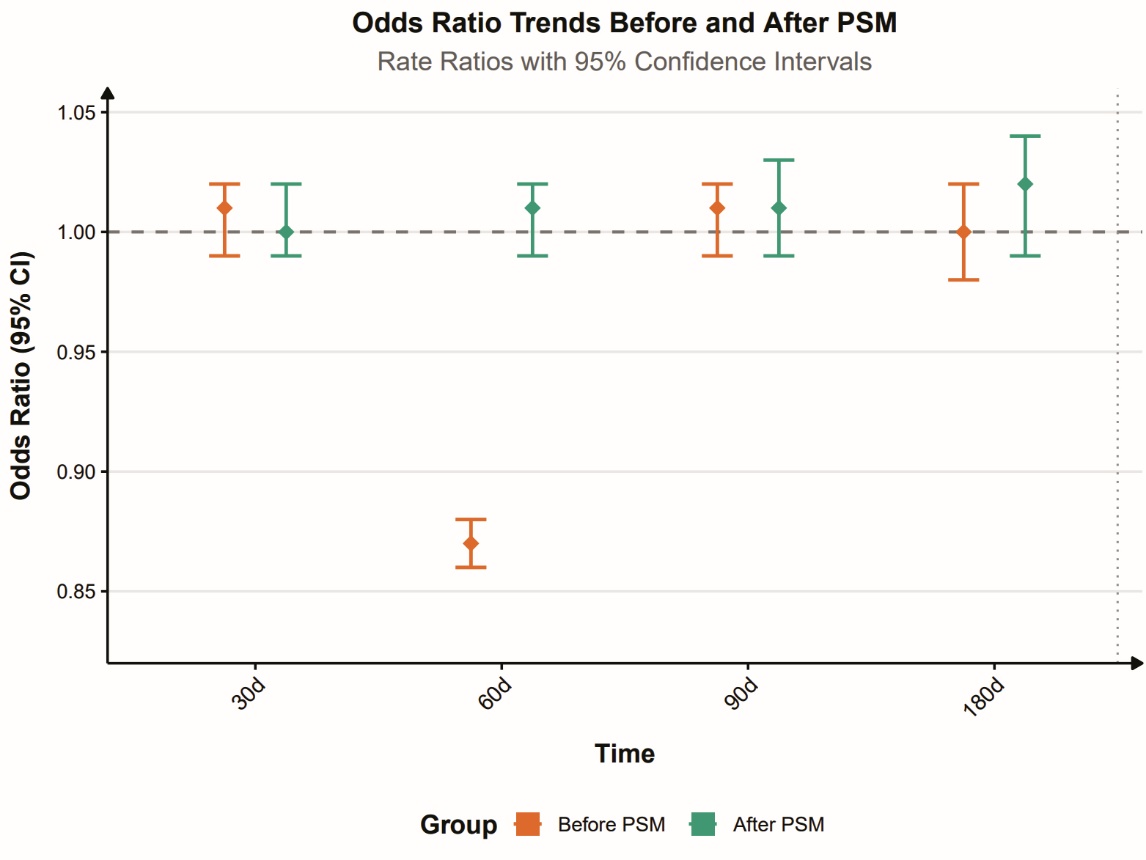
**

RR compares leadless pacemakers to TV-VVI pacemakers; RR < 1 indicates lower risk in the leadless group.

**Figure S2.** Love plot of covariate balance (|SMD|) before and after PSM.

**
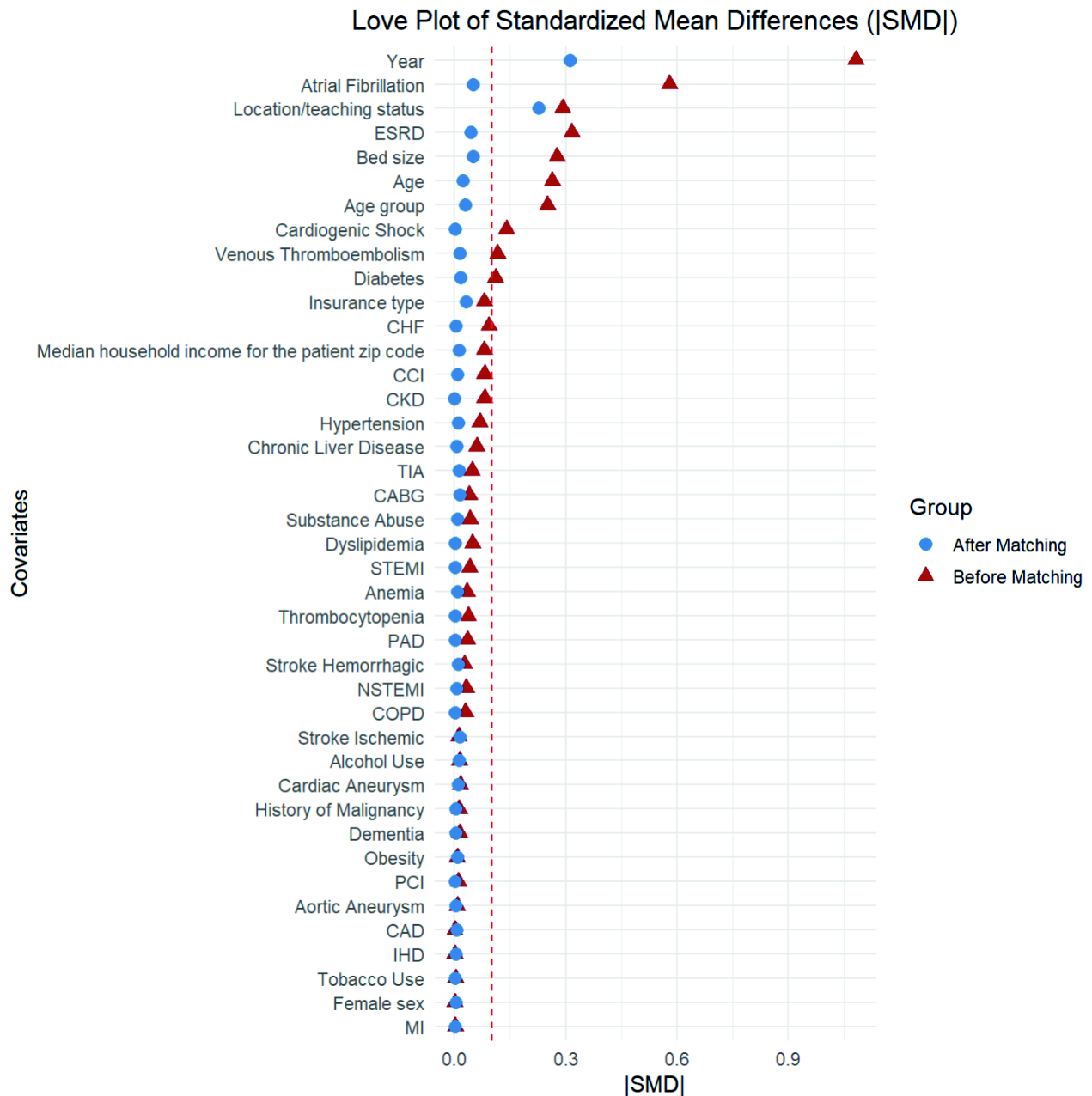
**

Each point represents the standardized mean difference (|SMD|) for a covariate. The red dashed vertical line indicates |SMD| = 0.1, a commonly used threshold for acceptable balance. Values < 0.1 suggest good balance between groups, whereas values > 0.1 indicate residual imbalance.

**Figure S3.** Breakdown of primary outcomes and logistic regression before and after PSM among patients with 60-day rehospitalization.


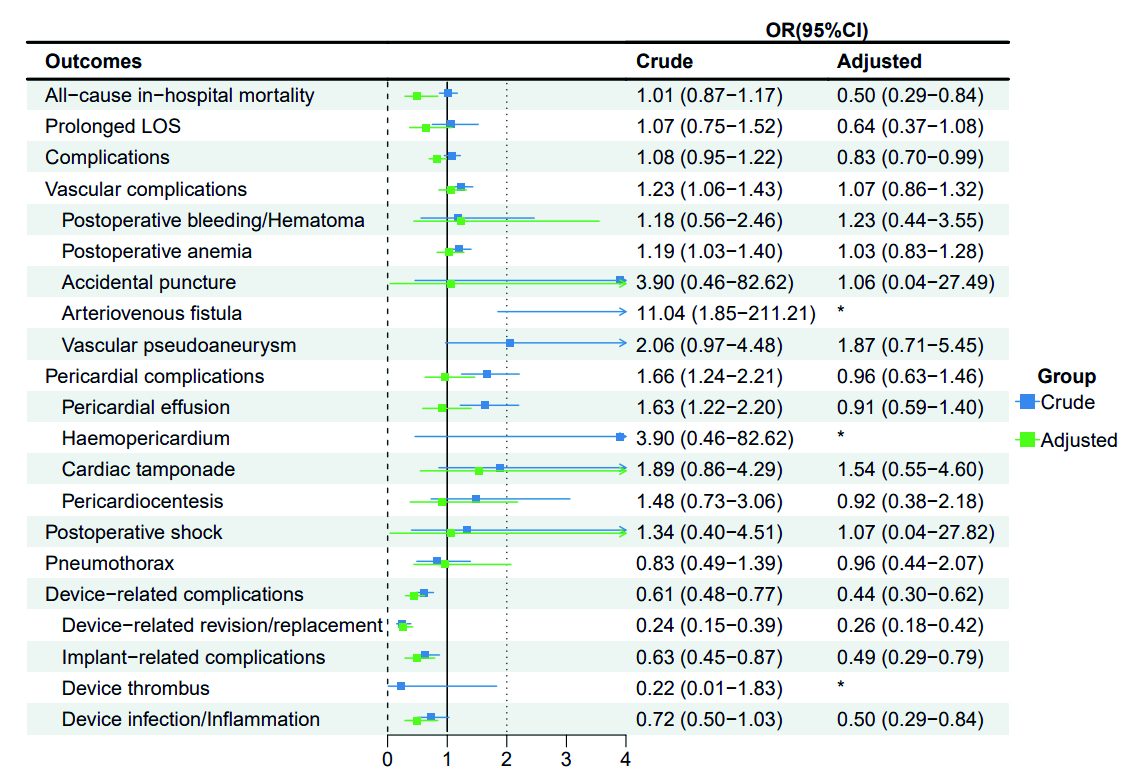


OR compares leadless pacemakers to TV-VVI pacemakers; OR < 1 indicates lower risk in the leadless group.

**Figure S4.** Breakdown of primary outcomes and logistic regression before and after PSM among patients with 90-day rehospitalization.


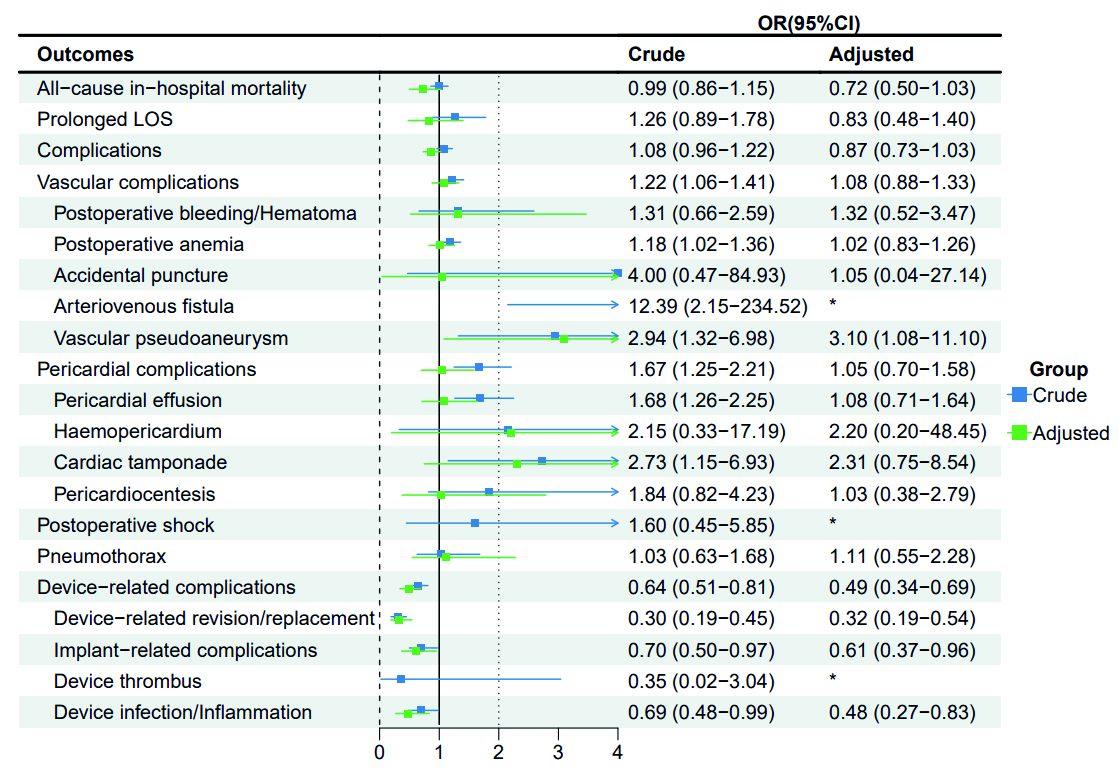


OR compares leadless pacemakers to TV-VVI pacemakers; OR < 1 indicates lower risk in the leadless group.

**Figure S5.** Breakdown of primary outcomes and logistic regression before and after PSM among patients with 180-day rehospitalization.


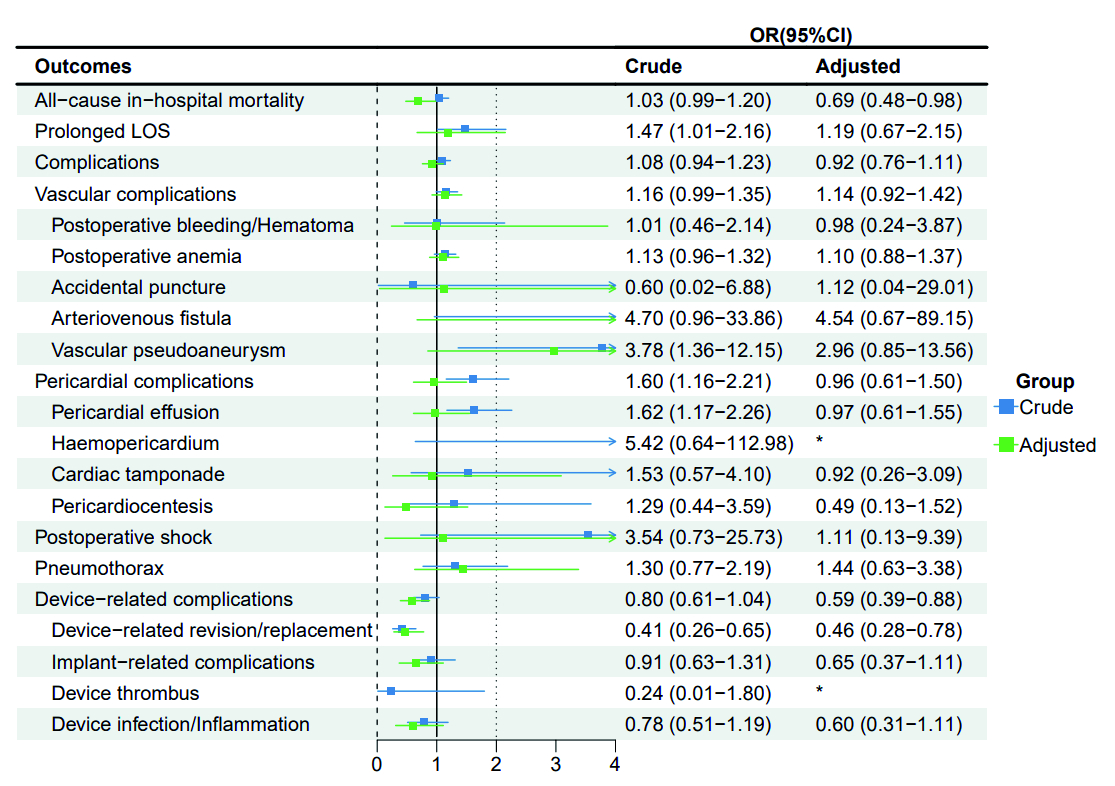


OR compares leadless pacemakers to TV-VVI pacemakers; OR < 1 indicates lower risk in the leadless group.

**Table S1.** Causes of 30-day readmission pacemaker implantation patients.

| **Cause/Type** | **Overall (%)**  **N=7383** | **TV-VVI (%)**  **N=4027** | **LPM (%)**  **N=3356** | **Risk ratio (RR)** |
| --- | --- | --- | --- | --- |
| **Cause of readmission** | | | | |
| Cardiac | 3015 (40.8) | 1769 (43.9) | 1246 (37.1) | 0.70 |
| Acute myocardial infarction | 104 (1.4) | 50 (1.2) | 54 (1.6) | 1.08 |
| Coronary artery disease / Ischemic heart disease | 48 (0.7) | 19 (0.5) | 29 (0.9) | 1.53 |
| Congestive heart failure | 1655 (22.4) | 997 (24.8) | 658 (19.6) | 0.66 |
| Arrhythmias/Conduction disorders | 371 (5.0) | 226 (5.6) | 145 (4.3) | 0.64 |
| Cardiac arrest/Cardiogenic shock | 9 (0.1) | 4 (0.1) | 5 (0.1) | 1.25 |
| Valvular heart disease | 159 (2.2) | 78 (1.9) | 81 (2.4) | 1.04 |
| Pericardial disease | 35 (0.5) | 13 (0.3) | 22 (0.7) | 1.69 |
| Pulmonary hypertension | 97 (1.3) | 48 (1.2) | 49 (1.5) | 1.02 |
| Device-related complications | 271 (3.7) | 153 (3.8) | 118 (3.5) | 0.77 |
| Cerebrovascular disease (cardio-related) | 266 (3.6) | 181 (4.5) | 85 (2.5) | 1.04 |
| Non-cardiac | 4368 (59.2) | 2258 (56.1) | 2110 (62.9) | 0.93 |
| Infections | 1055 (14.3) | 538 (13.4) | 517 (15.4) | 0.96 |
| Respiratory | 276 (3.7) | 154 (3.8) | 122 (3.6) | 0.79 |
| Peripheral and systemic vascular | 216 (2.9) | 114 (2.8) | 102 (3.0) | 0.47 |
| Renal | 230 (3.1) | 127 (3.2) | 103 (3.1) | 0.81 |
| Psychiatric | 57 (0.8) | 24 (0.6) | 33 (1.0) | 1.38 |
| Gastrointestinal | 517 (7.0) | 274 (6.8) | 243 (7.2) | 0.89 |
| Endocrine/Metabolic | 204 (2.8) | 85 (2.1) | 119 (3.5) | 1.40 |
| Central nervous system | 50 (0.7) | 27 (0.7) | 23 (0.7) | 0.85 |
| Hematological | 108 (1.5) | 61 (1.5) | 47 (1.4) | 0.77 |
| Neoplasms | 78 (1.1) | 44 (1.1) | 34 (1.0) | 0.77 |
| Musculoskeletal | 102 (1.4) | 55 (1.4) | 47 (1.4) | 0.86 |
| Dermatological | 111 (1.5) | 60 (1.5) | 51 (1.5) | 0.85 |
| Others | 1364 (18.5) | 695 (17.3) | 669 (19.9) | 0.96 |
| **Readmission Type** | | | | |
| Elective readmission | 881 (11.9) | 451 (11.2) | 430 (12.8) | 0.95 |
| Non-elective readmission | 6502 (88.1) | 3576 (88.8) | 2926 (87.2) | 0.82 |

December discharges were retained, as the aim was descriptive characterization of causes rather than estimation of precise readmission rates; descriptive only, no CIs; RR >1 indicates higher risk for LPM compared with TV-VVI.

**Table S2**. Breakdown of primary outcomes and logistic regression analysis before and after PSM among patients with 60-day rehospitalization.

| **Primary outcome** | **Before Propensity Matching** | | | **After Propensity Matching** | | | **Crude OR**  **(95% CI)** | **P-value** | **Adjusted OR**  **(95% CI)** | **P-value** |
| --- | --- | --- | --- | --- | --- | --- | --- | --- | --- | --- |
|  | **Overall**  **n=9483(%)** | **Leadless**  **n=4236(%)** | **TV-VVI**  **n=5247(%)** | **Overall**  **n=4078(%)** | **Leadless**  **n=2031(%)** | **TV-VVI**  **n=2067(%)** |  |  |  |  |
| All-cause in-hospital mortality | 858 (9.1) | 388 (9.2) | 470 (9.0) | 392 (9.6) | 179 (8.8) | 213 (10.3) | 1.01 (0.87-1.17) | 0.942 | 0.50 (0.29-0.84) | 0.011 |
| Prolonged LOS (≥30 days) | 140 (1.5) | 68 (1.6) | 72 (1.4) | 59 (1.4) | 23 (1.1) | 36 (1.7) | 1.07 (0.75-1.52) | 0.721 | 0.64 (0.37-1.08) | 0.103 |
| Complications | 1319 (14.0) | 623 (14.7) | 696 (13.3) | 574 (14.1) | 262 (12.9) | 312 (15.1) | 1.08 (0.95-1.22) | 0.245 | 0.83 (0.70-0.99) | 0.042 |
| Vascular complications | 848 (9.0) | 421 (9.9) | 427 (8.1) | 378 (9.3) | 193 (9.5) | 185 (9.0 | 1.23 (1.06-1.43) | 0.006 | 1.07 (0.86-1.32) | 0.557 |
| Postoperative bleeding/Hematoma | 31 (0.3) | 15 (0.4) | 16 (0.3) | 15 (0.4) | <11 | <11 | 1.18 (0.56-2.46) | 0.662 | 1.23 (0.44-3.55) | 0.693 |
| Postoperative anemia | 805 (8.5) | 395 (9.3) | 410 (7.8) | 356 (8.7) | 179 (8.8) | 177 (8.6) | 1.19 (1.03-1.40) | 0.019 | 1.03 (0.83-1.28) | 0.798 |
| Accidental puncture | <11 | <11 | <11 | <11 | <11 | <11 | 3.90 (0.46-82.62) | 0.254 | 1.06 (0.04-27.49) | 0.996 |
| Arteriovenous fistula | <11 | <11 | <11 | <11 | <11 | 0 | 11.04 (1.85-211.21) | 0.028 | * | 0.994 |
| Vascular pseudoaneurysm | 30 (0.3) | 18 (0.4) | 12 (0.2) | 17 (0.4) | 11 (0.4) | <11 | 2.06 (0.97-4.48) | 0.061 | 1.87 (0.71-5.45) | 0.218 |
| Pericardial complications | 215 (2.3) | 124 (2.9) | 91 (1.7) | 91 (2.2) | 44 (2.2) | 47 (2.3) | 1.66 (1.24-2.21) | <0.001 | 0.96 (0.63-1.46) | 0.845 |
| Pericardial effusion | 205 (2.2) | 118 (2.8) | 87 (1.7) | 85 (2.1) | 40 (2.0) | 45 (2.2) | 1.63 (1.22-2.20) | 0.001 | 0.91 (0.59-1.40) | 0.663 |
| Haemopericardium | <11 | <11 | <11 | <11 | <11 | 0 | 3.90 (0.46-82.62) | 0.254 | * | 0.996 |
| Cardiac tamponade | 28 (0.3) | 17 (0.4) | 11 (0.2) | 15 (0.4) | <11 | <11 | 1.89 (0.86-4.29) | 0.118 | 1.54 (0.55-4.60) | 0.414 |
| Pericardiocentesis | 34 (0.4) | 19 (0.4) | 15 (0.3) | 21 (0.5) | <11 | 11 (0.5) | 1.48 (0.73-3.06) | 0.272 | 0.92 (0.38-2.18) | 0.846 |
| Postoperative shock | 12 (0.1) | <11 | <11 | <11 | <11 | <11 | 1.34 (0.40-4.51) | 0.626 | 1.07 (0.04-27.82) | 0.959 |
| Pneumothorax | 65 (0.7) | 26 (0.6) | 39 (0.7) | 27 (0.7) | 13 (0.6) | 14 (0.7) | 0.83 (0.49-1.39) | 0.487 | 0.96 (0.44-2.07) | 0.921 |
| Device-related complications | 343 (3.6) | 128 (3.0) | 215 (4.1) | 147 (3.6) | 45 (2.2) | 102 (4.9) | 0.61 (0.48-0.77) | <0.001 | 0.44 (0.30-0.62) | <0.001 |
| Device-related revision/replacement | 131 (1.4) | 22 (0.5) | 108 (2.1) | 62 (1.5) | 13 (0.6) | 49 (2.3) | 0.24 (0.15-0.39) | <0.001 | 0.26 (0.18-0.42) | <0.001 |
| Implant related complication (Hemorrhage, Stenosis, Lead breakdown) | 171 (1.8) | 68 (1.6) | 103 (2.0) | 73 (1.8) | 24 (1.2) | 49 (2.4) | 0.63 (0.45-0.87) | 0.006 | 0.49 (0.29-0.79) | 0.004 |
| Device thrombus | <11 | <11 | <11 | <11 | 0 | <11 | 0.22 (0.01-1.83) | 0.199 | * | 0.998 |
| Device infection/Inflammation | 144 (1.4) | 57 (1.3) | 87 (1.7) | 63 (1.5) | 21 (1.0) | 42 (2.0) | 0.72 (0.50-1.03) | 0.072 | 0.50 (0.29-0.84) | 0.010 |

^a^Abbreviations: Crude OR, Odds ratio before propensity score matching; Adjusted OR: Odds ratio after propensity score matching; CI, confidence interval; PSM, propensity score matching; LOS, length of stay; TV-VVI, transvenous ventricular-inhibited pacing.

^b^Per HCUP policy, counts <11 are suppressed to protect privacy.

**Table S3**. Breakdown of primary outcomes and logistic regression analysis before and after PSM among patients with 90-day rehospitalization.

| **Primary outcome** | **Before Propensity Matching** | | | **After Propensity Matching** | | | **Crude OR**  **(95% CI)** | **P-value** | **Adjusted OR**  **(95% CI)** | **P-value** |
| --- | --- | --- | --- | --- | --- | --- | --- | --- | --- | --- |
|  | **Overall**  **n=10,283(%)** | **Leadless**  **n=4532(%)** | **TV-VVI**  **n=5751(%)** | **Overall**  **n=4423(%)** | **Leadless**  **n=2183(%)** | **TV-VVI**  **n=2240(%)** |  |  |  |  |
| All-cause in-hospital mortality | 939 (9.1) | 419 (9.2) | 520 (9.0) | 422 (9.5) | 193 (8.8) | 229 (10.2) | 0.99 (0.86-1.15) | 0.941 | 0.72 (0.50-1.03) | 0.071 |
| Prolonged LOS (≥30 days) | 144 (1.4) | 74 (1.6) | 70 (1.2) | 56 (1.3) | 25 (1.1) | 31 (1.4) | 1.26 (0.89-1.78) | 0.197 | 0.83 (0.48-1.40) | 0.478 |
| Complications | 1392 (13.5) | 652 (14.4) | 740 (12.9) | 613 (13.9) | 284 (13.0) | 329 (14.7) | 1.08 (0.96-1.22) | 0.195 | 0.87 (0.73-1.03) | 0.104 |
| Vascular complications | 917 (8.9) | 447 (9.9) | 470 (8.2) | 414 (9.4) | 212 (9.7) | 202 (9.0) | 1.22 (1.06-1.41) | <0.001 | 1.08 (0.88-1.33) | 0.441 |
| Postoperative bleeding/Hematoma | 36 (0.3) | 18 (0.4) | 18 (0.3) | 18 (0.4) | <11 | <11 | 1.31 (0.66-2.59) | 0.442 | 1.32 (0.52-3.47) | 0.564 |
| Postoperative anemia | 873 (8.5) | 419 (9.2) | 454 (7.9) | 390 (8.8) | 195 (8.9) | 195 (8.7) | 1.18 (1.02-1.36) | 0.028 | 1.02 (0.83-1.26) | 0.807 |
| Accidental puncture | <11 | <11 | <11 | <11 | <11 | <11 | 4.00 (0.47-84.93) | 0.246 | 1.05 (0.04-27.14) | 0.973 |
| Arteriovenous fistula | <11 | <11 | <11 | <11 | <11 | 0 | 12.39 (2.15-234.52) | 0.020 | * | 0.994 |
| Vascular pseudoaneurysm | 27 (0.3) | 18 (0.4) | 9 (0.2) | 16 (0.4) | 12 (0.5) | <11 | 2.94 (1.32-6.98) | 0.010 | 3.10 (1.08-11.10) | 0.049 |
| Pericardial complications | 218 (2.1) | 124 (2.7) | 94 (1.6) | 97 (2.2) | 49 (2.2) | 48 (2.1) | 1.67 (1.25-2.21) | <0.001 | 1.05 (0.70-1.58) | 0.805 |
| Pericardial effusion | 208 (2.0) | 119 (2.6) | 89 (1.5) | 90 (2.0) | 46 (2.1) | 44 (2.0) | 1.68 (1.26-2.25) | <0.001 | 1.08 (0.71-1.64) | 0.726 |
| Haemopericardium | <11 | <11 | <11 | <11 | <11 | <11 | 2.15 (0.33-17.19) | 0.418 | 2.20 (0.20-48.45) | 0.523 |
| Cardiac tamponade | 24 (0.2) | 16 (0.4) | <11 | 13 (0.3) | <11 | <11 | 2.73 (1.15-6.93) | 0.027 | 2.31 (0.75-8.54) | 0.164 |
| Pericardiocentesis | 26 (0.3) | 15 (0.3) | 11 (0.2) | 16 (0.4) | <11 | <11 | 1.84 (0.82-4.23) | 0.143 | 1.03 (0.38-2.79) | 0.961 |
| Postoperative shock | 11 (0.1) | <11 | <11 | <11 | <11 | 0 | 1.60 (0.45-5.85) | 0.457 | * | 0.998 |
| Pneumothorax | 72 (0.7) | 32 (0.7) | 40 (0.7) | 31 (0.7) | 16 (0.7) | 15 (0.7) | 1.03 (0.63-1.68) | 0.905 | 1.11 (0.55-2.28) | 0.768 |
| Device-related complications | 348 (3.4) | 134 (3.0) | 214 (3.7 | 147 (3.3) | 48 (2.2) | 99 (4.4) | 0.64 (0.51-0.81) | <0.001 | 0.49 (0.34-0.69) | <0.001 |
| Device-related revision/replacement | 141 (1.4) | 27 (0.6) | 114 (2.0) | 66 (1.5) | 16 (0.7) | 50 (2.2) | 0.30 (0.19-0.45) | <0.001 | 0.32 (0.19-0.54) | <0.001 |
| Implant related complication (Hemorrhage, Stenosis, Lead breakdown) | 173 (1.7) | 73 (1.6) | 100 (1.7) | 75 (1.7) | 28 (1.3) | 47 (2.1) | 0.70 (0.50-0.97) | 0.032 | 0.61 (0.37-0.96) | 0.037 |
| Device thrombus | <11 | <11 | <11 | <11 | 0 | <11 | 0.35 (0.02-3.04) | 0.383 | * | 0.997 |
| Device infection/Inflammation | 140 (1.4) | 55 (1.2) | 85 (1.5) | 59 (1.3) | 19 (0.9) | 40 (1.8) | 0.69 (0.48-0.99) | 0.045 | 0.48 (0.27-0.83) | 0.010 |

^a^Abbreviations: Crude OR, Odds ratio before propensity score matching; Adjusted OR: Odds ratio after propensity score matching; CI, confidence interval; PSM, propensity score matching; LOS, length of stay; TV-VVI, transvenous ventricular-inhibited pacing.

^b^Per HCUP policy, counts <11 are suppressed to protect privacy.

**Table S4**. Breakdown of primary outcomes and logistic regression analysis before and after PSM among patients with 180-day rehospitalization.

| **Primary outcome** | **Before Propensity Matching** | | | **After Propensity Matching** | | | **Crude OR**  **(95% CI)** | **P-value** | **Adjusted OR**  **(95% CI)** | **P-value** |
| --- | --- | --- | --- | --- | --- | --- | --- | --- | --- | --- |
|  | **Overall**  **n=9021(%)** | **Leadless**  **n=3829(%)** | **TV-VVI**  **n=5192(%)** | **Overall**  **n=3796(%)** | **Leadless**  **n=1823(%)** | **TV-VVI**  **n=1973(%)** |  |  |  |  |
| All-cause in-hospital mortality | 810 (9.0) | 352 (9.2) | 458 (8.8) | 377 (8.5) | 171 (7.8) | 206 (9.2) | 1.03 (0.99-1.20) | 0.671 | 0.69 (0.48-0.98) | 0.045 |
| Prolonged LOS (≥30 days) | 122 (1.4) | 66 (1.7) | 56 (1.1) | 46 (1.0) | 24 (1.1) | 22 (1.0) | 1.47 (1.01-2.16) | 0.045 | 1.19 (0.67-2.15) | 0.552 |
| Complications | 1147 (12.7) | 517 (13.5) | 630 (12.1) | 505 (11.4) | 234 (10.7) | 271 (12.1) | 1.08 (0.94-1.23) | 0.262 | 0.92 (0.76-1.11) | 0.399 |
| Vascular complications | 796 (8.8) | 365 (9.5) | 431 (8.3) | 359 (8.1) | 183 (8.4) | 176 (7.9) | 1.16 (0.99-1.35) | 0.057 | 1.14 (0.92-1.42) | 0.238 |
| Postoperative bleeding/Hematoma | 30 (0.3) | 13 (0.3) | 17 (0.3) | <11 | <11 | <11 | 1.01 (0.46-2.14) | 0.981 | 0.98 (0.24-3.87) | 0.987 |
| Postoperative anemia | 763 (8.5) | 346 (9.0) | 417 (8.0) | 343 (7.8) | 172 (7.9) | 171 (7.6) | 1.13 (0.96-1.32) | 0.131 | 1.10 (0.88-1.37) | 0.411 |
| Accidental puncture | <11 | <11 | <11 | <11 | <11 | <11 | 0.60 (0.02-6.88) | 0.686 | 1.12 (0.04-29.01) | 0.935 |
| Arteriovenous fistula | <11 | <11 | <11 | <11 | <11 | <11 | 4.70 (0.96-33.86) | 0.071 | 4.54 (0.67-89.15) | 0.177 |
| Vascular pseudoaneurysm | 17 (0.2) | 12 (0.3) | <11 | 11 (0.2) | <11 | <11 | 3.78 (1.36-12.15) | 0.015 | 2.96 (0.85-13.56) | 0.110 |
| Pericardial complications | 171 (1.9) | 91 (2.4) | 80 (1.5) | 79 (1.8) | 37 (1.7) | 42 (1.9) | 1.60 (1.16-2.21) | 0.004 | 0.96 (0.61-1.50) | 0.847 |
| Pericardial effusion | 162 (1.8) | 87 (2.3) | 75 (1.4) | 72 (1.6) | 34 (1.6) | 38 (1.7) | 1.62 (1.17-2.26) | 0.004 | 0.97 (0.61-1.55) | 0.905 |
| Haemopericardium | <11 | <11 | <11 | <11 | <11 | 0 | 5.42 (0.64-112.98) | 0.154 | * | 0.997 |
| Cardiac tamponade | 18 (0.2) | <11 | <11 | 11 (0.2) | <11 | <11 | 1.53 (0.57-4.10) | 0.392 | 0.92 (0.26-3.09) | 0.895 |
| Pericardiocentesis | 16 (0.2) | <11 | <11 | 13 (0.3) | <11 | <11 | 1.29 (0.44-3.59) | 0.627 | 0.49 (0.13-1.52) | 0.240 |
| Postoperative shock | <11 | <11 | <11 | <11 | <11 | <11 | 3.54 (0.73-25.73) | 0.144 | 1.11 (0.13-9.39) | 0.919 |
| Pneumothorax | 63 (0.7) | 31 (0.8) | 32 (0.6) | 23 (0.5) | 13 (0.6) | <11 | 1.30 (0.77-2.19) | 0.327 | 1.44 (0.63-3.38) | 0.390 |
| Device-related complications | 260 (2.9) | 110 (2.9) | 150 (2.9) | 103 (2.3 | 37 (1.7) | 66 (2.9) | 0.80 (0.61-1.04) | 0.099 | 0.59 (0.39-0.88) | 0.011 |
| Device-related revision/replacement | 102 (1.1) | 24 (0.6) | 78 (1.5) | 46 (1.2) | 14 (0.8) | 32 (1.4) | 0.41 (0.26-0.65) | <0.001 | 0.46 (0.28-0.78) | 0.019 |
| Implant related complication (Hemorrhage, Stenosis, Lead breakdown) | 135 (1.5) | 64 (1.7) | 71 (1.4) | 57 (1.3) | 22 (1.0) | 35 (1.6) | 0.91 (0.63-1.31) | 0.605 | 0.65 (0.37-1.11) | 0.119 |
| Device thrombus | <11 | <11 | <11 | <11 | 0 | <11 | 0.24 (0.01-1.80) | 0.220 | * | 0.997 |
| Device infection/Inflammation | 104 (1.2) | 42 (1.1) | 62 (1.2) | 42 (0.9) | 15 (0.7) | 27 (1.2) | 0.78 (0.51-1.19) | 0.256 | 0.60 (0.31-1.11) | 0.111 |

^a^Abbreviations: Crude OR, Odds ratio before propensity score matching; Adjusted OR: Odds ratio after propensity score matching; CI, confidence interval; PSM, propensity ^b^score matching; LOS, length of stay; TV-VVI, transvenous ventricular-inhibited pacing.

Per HCUP policy, counts <11 are suppressed to protect privacy.

**Table S5.** Comparison of readmission rates before and after PSM.

| Readmission | Group | Before Propensity Matching | | | After Propensity Matching | | |
| --- | --- | --- | --- | --- | --- | --- | --- |
|  |  | No. of Patients | No. of Readmissions | Readmission Rate (%) | No. of Patients | No. of Readmissions | Readmission Rate (%) |
| 30-day Readmission | Overall | 45,599 | 7082 | 15.5 | 19,344 | 3046 | 15.8 |
|  | Leadless | 20,273 | 3216 | 15.9 | 9574 | 1519 | 15.9 |
|  | TV-VVI | 25,326 | 3866 | 15.3 | 9770 | 1527 | 15.6 |
| 60-day Readmission | Overall | 41,483 | 9483 | 22.9 | 17,622 | 4098 | 23.3 |
|  | Leadless | 23,226 | 4236 | 18.2 | 8644 | 2031 | 23.5 |
|  | TV-VVI | 18,259 | 5247 | 28.7 | 8978 | 2067 | 23.0 |
| 90-day Readmission | Overall | 37,249 | 10,283 | 27.6 | 15,832 | 4423 | 27.9 |
|  | Leadless | 16,251 | 4532 | 27.9 | 7696 | 2183 | 28.4 |
|  | TV-VVI | 20,998 | 5751 | 27.4 | 8136 | 2240 | 27.5 |
| 180-day Readmission | Overall | 24,820 | 9021 | 36.4 | 10,564 | 3796 | 35.9 |
|  | Leadless | 10,496 | 3829 | 36.5 | 5002 | 1823 | 36.5 |
|  | TV-VVI | 14,324 | 5192 | 36.2 | 5562 | 1973 | 35.5 |
